# Supplementary material for: Synthetic aptamer mechanoreceptors enable cell-specific force sensing and temporal control via DNA circuits
Source: Nat Commun. 2026 Mar 15;17:2492. doi: 10.1038/s41467-026-70765-w (PMC12992700; doi:10.1038/s41467-026-70765-w)
Supplement: Supplementary file 1 — Supplementary Information [file 41467_2026_70765_MOESM1_ESM.pdf]

Supplementary Information

Synthetic Aptamer Mechanoreceptors Enable Cell-Specific Force Sensing and Temporal Control via DNA Circuits

Tao Xu[1], Soumya Sethi[1], Christoph Drees[1], and Andreas Walther[1,2]*

Affiliations

[1] Life-Like Materials and Systems, University of Mainz, Duesbergweg 10-14, Mainz 55128, Germany.

[2] Max Planck Institute for Polymer Research, Ackermannweg 10, 55128 Mainz, Germany.

*Corresponding author. Email: andreas.walther@uni-mainz.de

Supplementary Tables

Supplementary Table 1. DNA sequences for the oligonucleotides with their names, sequence codes, and modifications.

Name	Sequence 5'-3'	Modification
Unzipping mode	AGT CGT ATT ACC GCG TTT	5' Rhodamin Red - X<br>3' Biotin-TEG
Shearing mode	TTT 8AG TCG TAT TA CCGCG	5' Biotin-TEG<br>8 dT-Rhodamine Red-X
AS1411 strand	GGT GGT GGT GGT TGT GGT GGT GGT GGC GCG GTA ATA CGA CT	3' BHQ2
AS1411 strand with Atto 488	TTG GTG GTG GTG GTT GTG GTG GTG GTG GCG CGG TAA TAC GAC T	5' Atto 488
Random strand	CCT CCT CCT CCT TCT CCT CCT CCT CCC GCG GTA ATA CGA CT	3' BHQ2
Null strand	CGC GGT AAT ACG ACT	3' BHQ2
TB-5 strand	TTT TTA AGC CCA CTC CTC TGT GGG GGG CGA ACA ACA AGG CAG TCG TGC CAT GCG CGG TAA TAC GAC T	3' BMNQ1
DBCO strand [a]	TTC GCG GTA ATA CGA CT	5' DBCO<br>3' BHQ2
Sgc8 strand	ATC TAA CTG CTG CGC CGC CGG GAA AAT ACT GTA CGG TTA GAC GCG GTA ATA CGA CT	3' BHQ2
MUC1 strand	GCA GTT GAT CCT TTG GAT ACC CTG GCG CGG TAA TAC GAC T	3' BHQ2
SYL3c strand	CAC TAC AGA GGT TGC GTC TGT CCC ACG TTG TCA TGG GGG GTT GGC CTG CGC GGT AAT ACG ACT	3' BHQ2
DNA Blocker strand	TAC GGC GAG ACA CCA CCA CCA CCA CAA CCA CCA CCA CC	3' BHQ1
Activator strand	GGT GGT GGT GGT TGT GGT GGT GGT GGT GTC TCG CCG TA	
RNA blocker strand	CCA CCA CCA CCA CAA CCA CCA CCA CC	3' BHQ1
Random RNA strand	GCA UUU CGU CUU AAU AUA GCA UGU CA	
Random DNA strand	TGA CAT GCT ATA TTA AGA CGA AAT GC	
AS1411 strand without quencher	GGT GGT GGT GGT TGT GGT GGT GGT GGC GCG GTA ATA CGA CT	
MUC1 strand without quencher	GCA GTT GAT CCT TTG GAT ACC CTG GCG CGG TAA TAC GAC T	
Sgc8 strand without quencher	ATC TAA CTG CTG CGC CGC CGG GAA AAT ACT GTA CGG TTA GAC GCG GTA ATA CGA CT	
SYL3c strand without quencher	CAC TAC AGA GGT TGC GTC TGT CCC ACG TTG TCA TGG GGG GTT GGC CTG CGC GGT AAT ACG ACT	
DBCO strand without quencher	TT CGCGG TAA TAC GAC T	5' DBCO

[a] We used click chemistry to conjugate cRGDfK-N_3 to DBCO strand for obtaining RGD strand based on our reported method.[1]

Supplementary Table 2. Surface density of aptamer MPs and RGD molecules. n = 18 from 3 replicates (mean ± s.d.).

	**MP density (molecule/μm^2)**	**RGD-bio density (molecule/μm^2)[a]**
AS1411 unzipping	4329±394	1297±155
Sgc8 unzipping	4480±239	1320±159
MUC1 S2.2 unzipping	4040±216	1322±101
SYL3c unzipping	4072±166	1356±134
AS1411 shearing	4141±269	1254±82
Sgc8 shearing	3918±609	1321±61
RGD unzipping	3495±201	1374±92
RGD shearing	3581±241	1318±155

Supplementary Table 3. Reaction rate constant of SDR and RNA-RNase H modules. To understand the reconfiguration efficiency and tunability of different DNRs, we quantified the surface reconfiguration kinetics. The data were analyzed assuming pseudo-first-order kinetics and fitted using one phase exponential decay function. For the SDR module, reactivation with 200 nM DNA activator yields a reconfiguration rate of 2.14×10^{-2} min^{-1}. The RNA-RNase H module enables degradation rate tuning by adjusting the initial RNase H concentration, resulting in accelerated (100 U/mL, 5.26×10^{-2} min^{-1}) or decelerated (10 U/mL, 0.71×10^{-2} min^{-1}) reconfiguration kinetics. Moreover, at a fixed RNase H concentration (10 U/mL), the RNA-RNase H module allows the introduction of non-enzymatic RNA/DNA decoys, providing an additional orthogonal layer of regulation.

	k **(×10^{-2} min^{-1})**	**R^2**
SDR 200 nM DNA activator	2.14	0.9926
RNase H 100U/mL	5.26	0.9925
RNase H 10U/mL	0.71	0.9842
RNase H 10U/mL+1 nM rdm RNA/DNA decoy	0.73	0.9782
RNase H 10U/mL+10 nM rdm RNA/DNA decoy	0.42	0.9603
RNase H 10U/mL+100 nM rdm RNA/DNA decoy	0.11	0.9831

Supplementary Figures

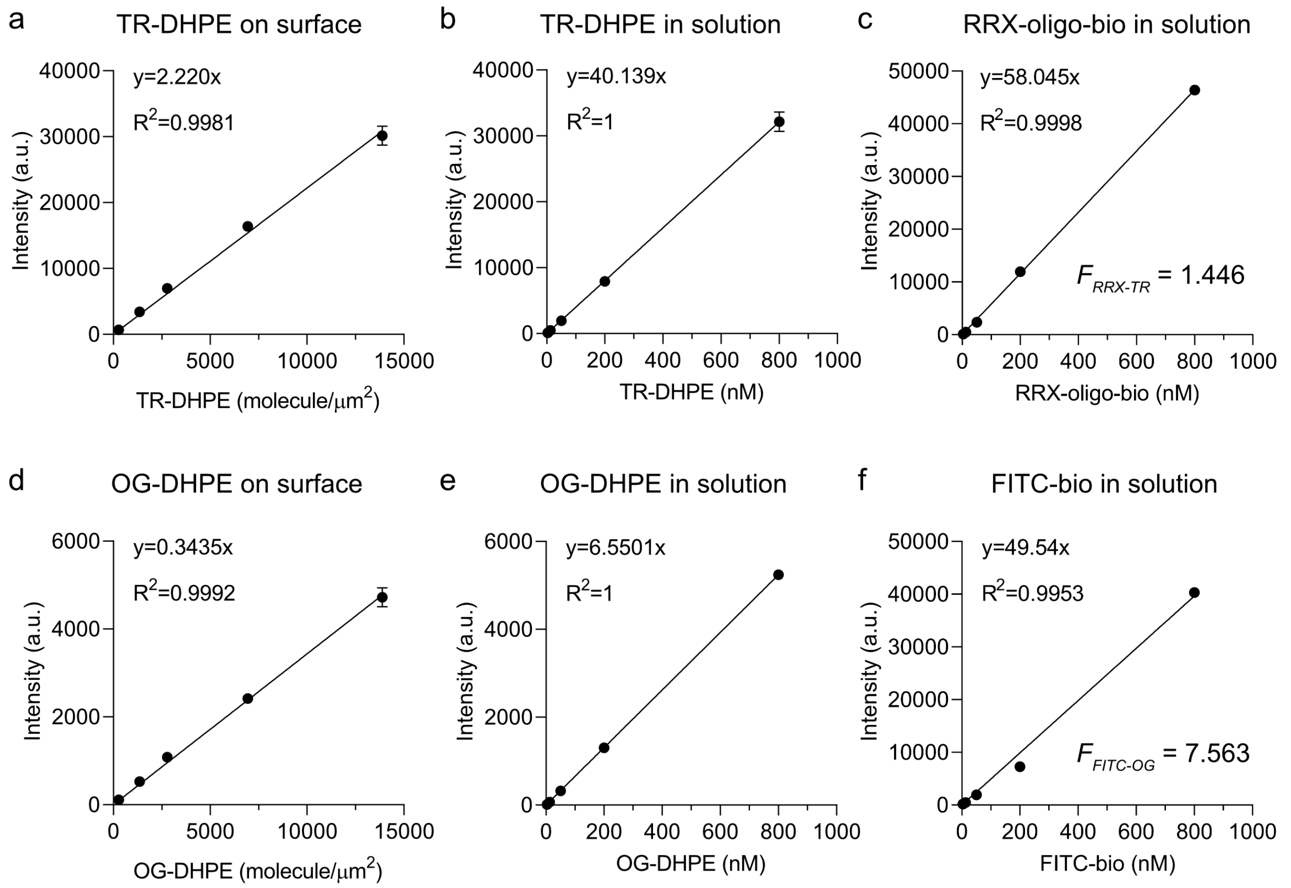

Supplementary Figure 1. Quantification of surface density of aptamer MPs and RGD molecules on the surface. **a-c** Standard curves for quantifying density of aptamer MPs. **a** SLB calibration: Intensity of Texas Red DHPE (Texas Red™ 1,2-dihexadecanoyl-sn-glycero-3-phosphoethanolamine, triethylammonium salt) to the number of molecules on the surface. n = 15 from 3 replicates. **b-c** F-factor plot estimation using concentrations of TR-DHPE-derived SUVs (**b**) and RRX-labelled oligonucleotide in solution (**c**) to compare the fluorescence intensity with density. The ratio of the calibration curve slopes was used to determine the "*F* factor" for the labelled oligonucleotide and the SUV samples. n = 15 from 3 replicates. **d-f** Standard curves for quantifying density of RGD molecules. Direct quantification of the pristine c[RGDfK(Biotin)] molecule is unfeasible due to its lack of a fluorophore. Using a fluorophore-conjugated RGD-biotin variant may distort the actual RGD density due to the increase in size and steric hindrance. Thus, we chose FITC-biotin (Fluorescein-5(6)-biotinamidohexanoylamidopentylthiourea, Mw 831.01 g/mol) as a proxy to estimate the RGD density, given its similar molecular weight to c[RGDfK(Biotin)] (Mw 829.98 g/mol). It should be noted that this approach ignores potential effects arising from differences in charge and hydrophilicity/hydrophobicity. **d** SLB calibration: Intensity of Oregon Green 488 DHPE (Oregon Green™ 488 1,2-Dihexadecanoyl-*sn*-Glycero-3-Phosphoethanolamine) to the number of molecules on the surface. n = 15 from 3 replicates. **e-f** F-factor plot estimation using concentrations of OG-DHPE-derived SUVs (**e**) and FITC-biotin in solution (**f**) to compare the fluorescence intensity with density. The ratio of the calibration curve slopes was used to determine the "*F* factor" for the labelled oligonucleotide and the SUV samples. n = 12 from 3 replicates. All graphs are presented as mean ± s.d. a.u., arbitrary units. Source data for this figure is available in the Source Data file.

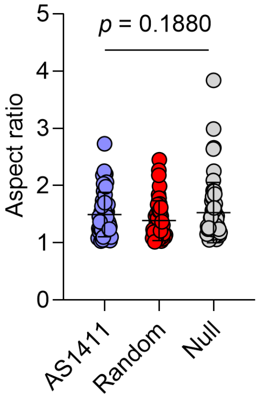

Supplementary Figure 2. Quantification of aspect ratio of HeLa cells on AS1411 MP surfaces. n = 54 cells from 3 replicates (mean ± s.d.). Statistics: Kruskal-Wallis test with Dunn's multiple comparisons. Source data for this figure is available in the Source Data file.

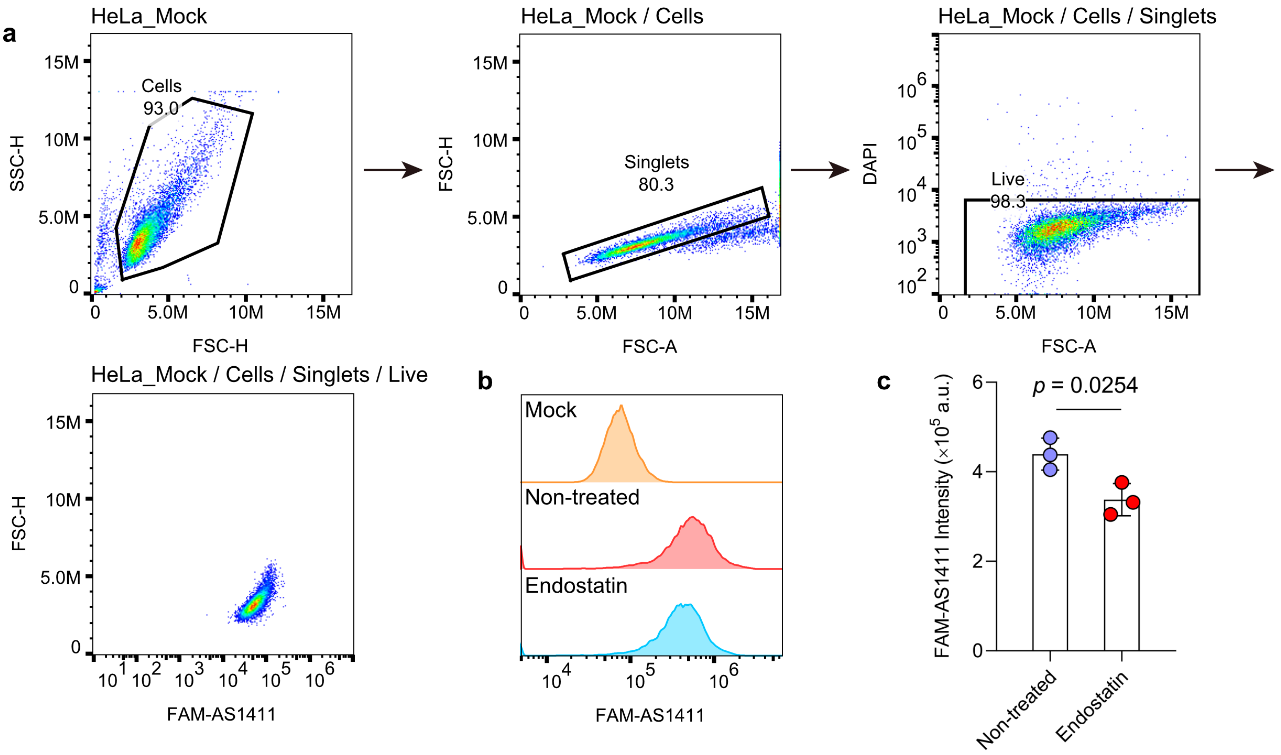

Supplementary Figure 3. a Flow cytometry gating strategy. **b** Representative flow cytometry histogram showing HeLa cells incubated with 2.5 µM FAM-AS1411 at 4 °C for 60 min, either in the absence or presence of 25 µg/mL endostatin. The mock control represents cells without FAM-AS1411, indicating cellular autofluorescence. The image is representative of 3 replicates. **c** Median fluorescence intensity of FAM-AS1411 in HeLa cells after endostatin treatment. Endostatin significantly inhibits AS1411 binding to HeLa cells. n = 3 replicates (mean ± s.d.). a.u., arbitrary units. Statistics: unpaired two-tailed Student's *t*-test. Source data for this figure is available in the Source Data file.

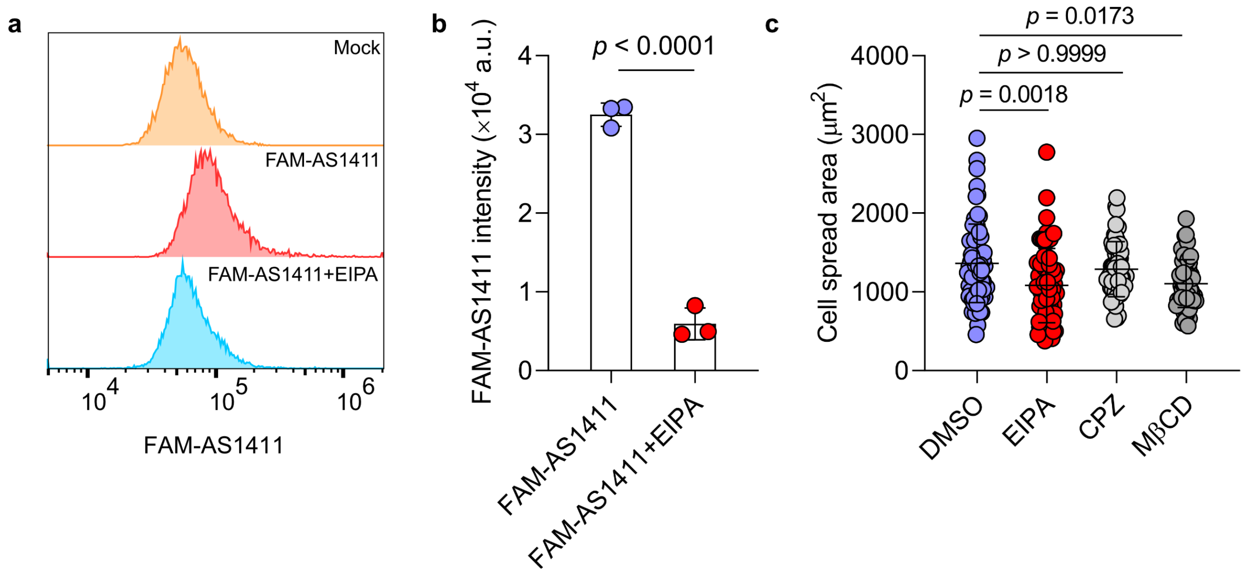

Supplementary Figure 4. a Representative flow cytometry histogram showing uptake of 500 nM FAM-AS1411 by HeLa cells at 37 °C for 4 h, either in the absence or presence of 100 μM EIPA. The mock control represents cells without FAM-AS1411, indicating cellular autofluorescence. The image is representative of 3 replicates. **b** Median fluorescence intensity of FAM-AS1411 in HeLa cells after EIPA treatment. EIPA significantly inhibits AS1411 internalization by HeLa cells. n = 3 replicates. Statistics: unpaired two-tailed Student's *t*-test. **c** Quantification of cell spreading area when HeLa cells were treated with different internalization inhibitors on AS1411 MP surfaces. n = 70, 70, 54, 54 cells from 3 replicates. Statistics: Kruskal-Wallis test with Dunn's multiple comparisons. Both graphs are presented as mean ± s.d. a.u., arbitrary units. Source data for this figure is available in the Source Data file.

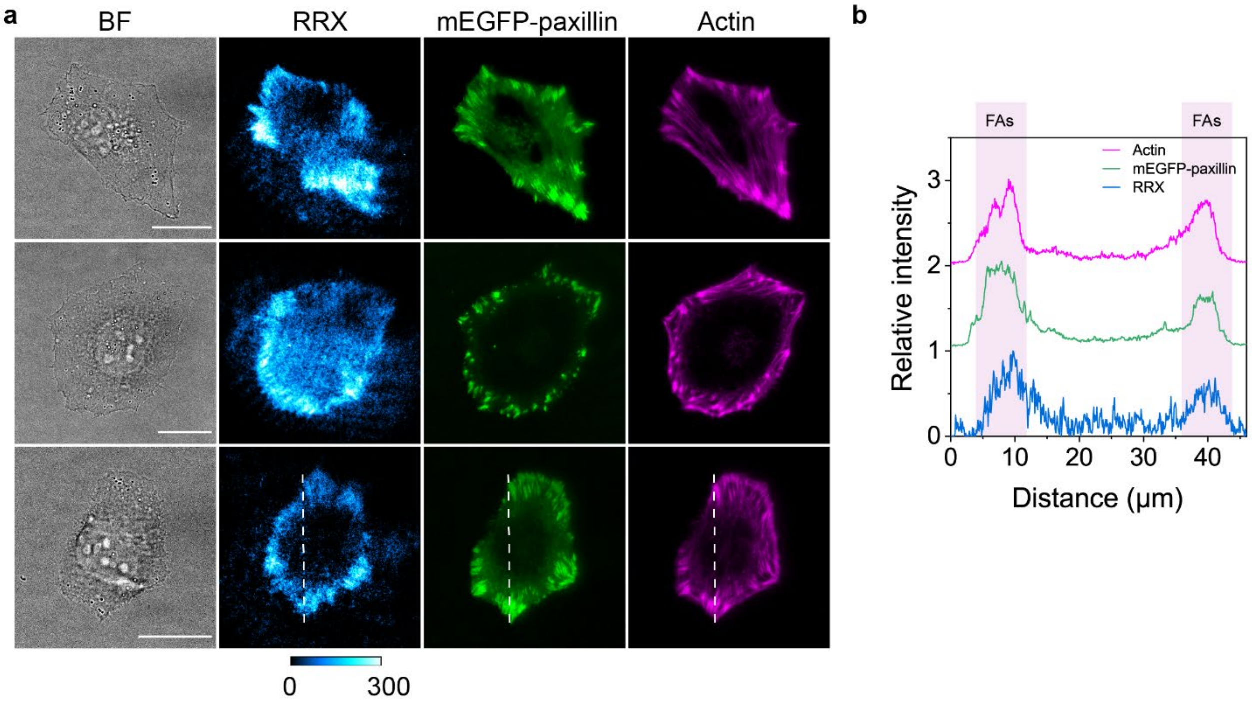

Supplementary Figure 5. a Representative microscopy imaging of brightfield, mechanosiganls, mEGFP-paxillin, and actin of HeLa cells incubated on AS1411 MP surfaces. **b** Line profile shows the intensity profiles of mEGFP-paxillin, actin and mechanosiganls along the white line marked in **a**. Nucleolin-generated forces are clearly localized at peripheral FA sites marked by paxillin. The images are representative of 3 replicates. Scale bars = 20 μm. Source data for this figure is available in the Source Data file.

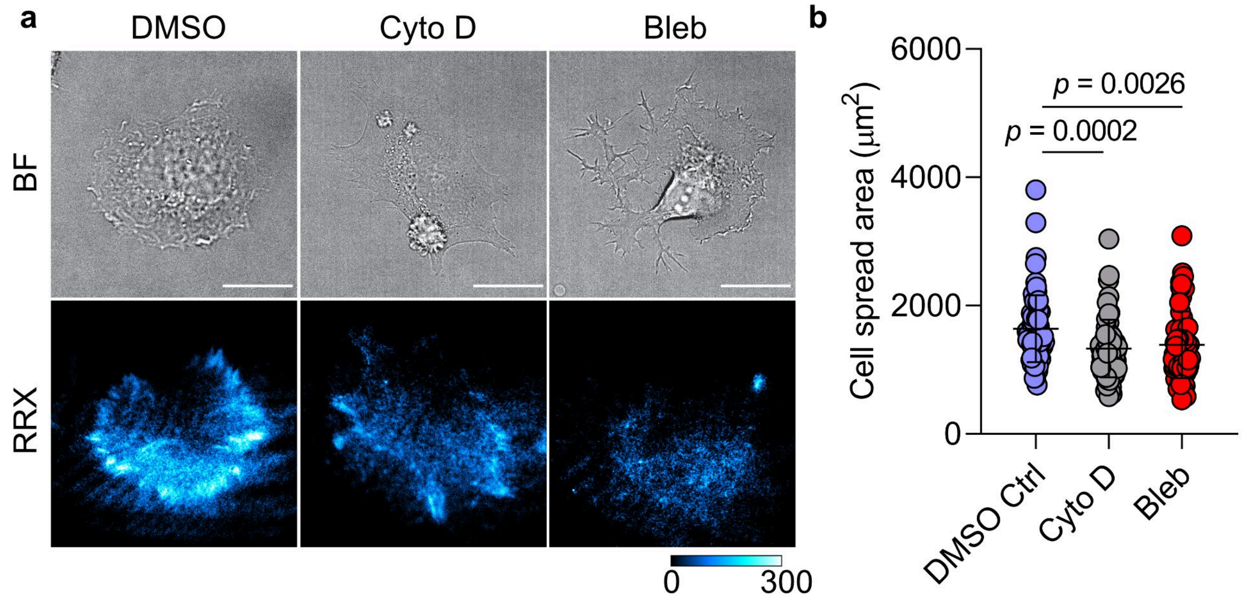

Supplementary Figure 6. a Representative brightfield and fluorescence images of HeLa cells treated with different cytoskeleton inhibitors on AS1411 MP surfaces. The image is representative of 3 replicates. **b** Quantification of cell spreading area when HeLa cells were treated with different cytoskeleton inhibitors on AS1411 MP surfaces. Both cytochalasin D and blebbistatin reduce cell spreading area. n = 72 cells from 3 replicates (mean ± s.d.). Statistics: Kruskal-Wallis test with Dunn's multiple comparisons. Scale bars = 20 µm. Source data for this figure is available in the Source Data file.

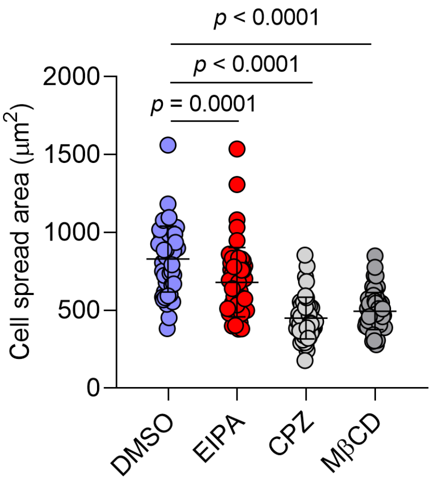

Supplementary Figure 7. Quantification of cell spreading area when HepG2 cells were treated with different internalization inhibitors on Sgc8 MP surfaces. n = 54 cells from 3 replicates (mean ± s.d.). Statistics: One-way ANOVA with Bonferroni post-hoc tests. Source data for this figure is available in the Source Data file.

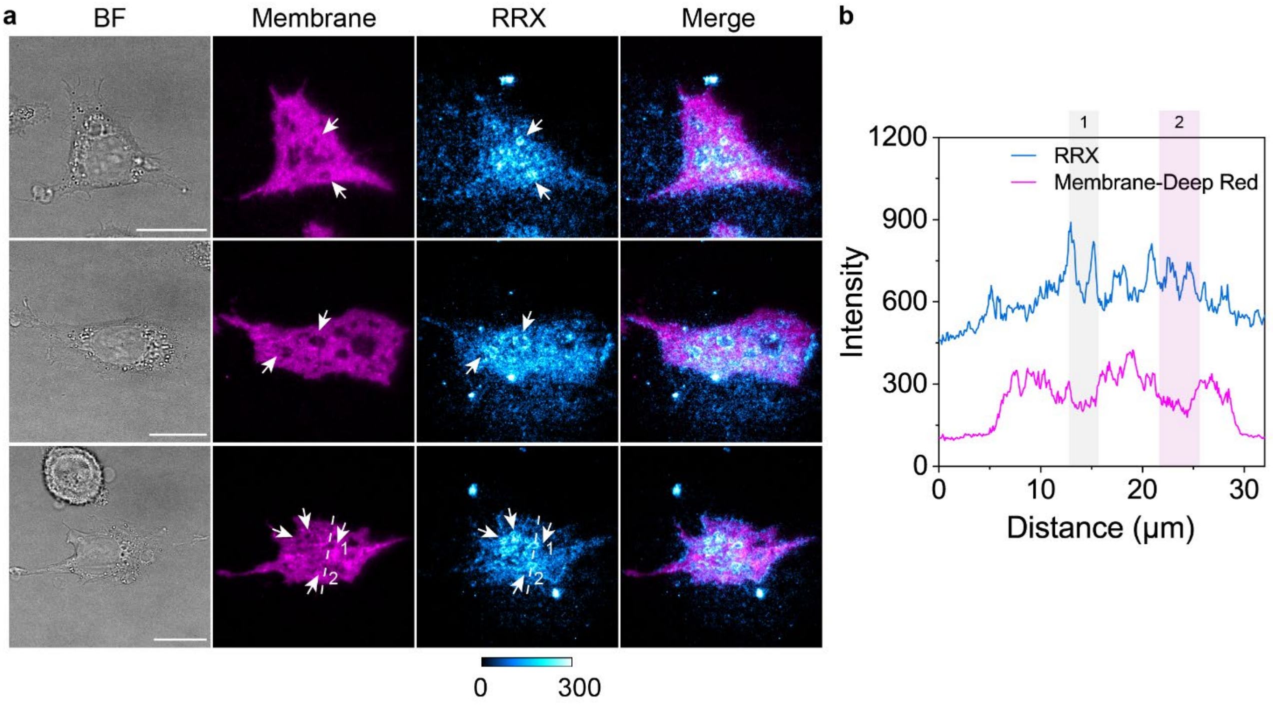

Supplementary Figure 8. a Representative microscopy imaging of brightfield, plasma membrane, and mechanosignals of HepG2 cells incubated on Sgc8 MP surfaces. The images are representative of 3 replicates. **b** Line profile shows the intensity profiles of plasma membrane and mechanosignals along the white line marked in **b**. Mechanosignals are localized at the inner edge of membrane invaginations. Scale bars = 20 μm. Source data for this figure is available in the Source Data file.

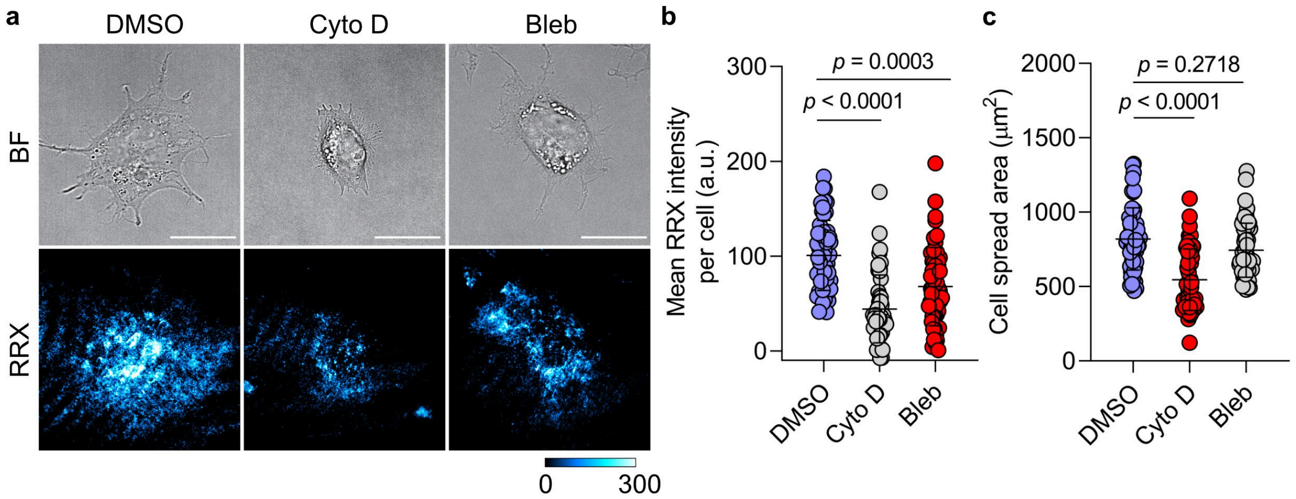

Supplementary Figure 9. a Representative brightfield and fluorescent images of HepG2 cells treated with different cytoskeleton inhibitors on Sgc8 MP surfaces. The image is representative of 3 replicates. **b-c** Quantification of mean fluorescence intensity per cell (**b**) and cell spreading area (**c**) when HepG2 cells were treated with different cytoskeleton inhibitors on Sgc8 MP surfaces. Cytochalasin D (actin polymerization inhibitor) exerts a stronger inhibitory effect than blebbistatin (myosin II inhibitor), indicating that actin polymerization plays a more important role than actomyosin contractility in PTK7-mediated force generation. n = 54 cells from 3 replicates. Statistics (b, c): Kruskal-Wallis test with Dunn's multiple comparisons. Both graphs are presented as mean ± s.d. a.u., arbitrary units. Scale bars = 20 μm. Source data for this figure is available in the Source Data file.

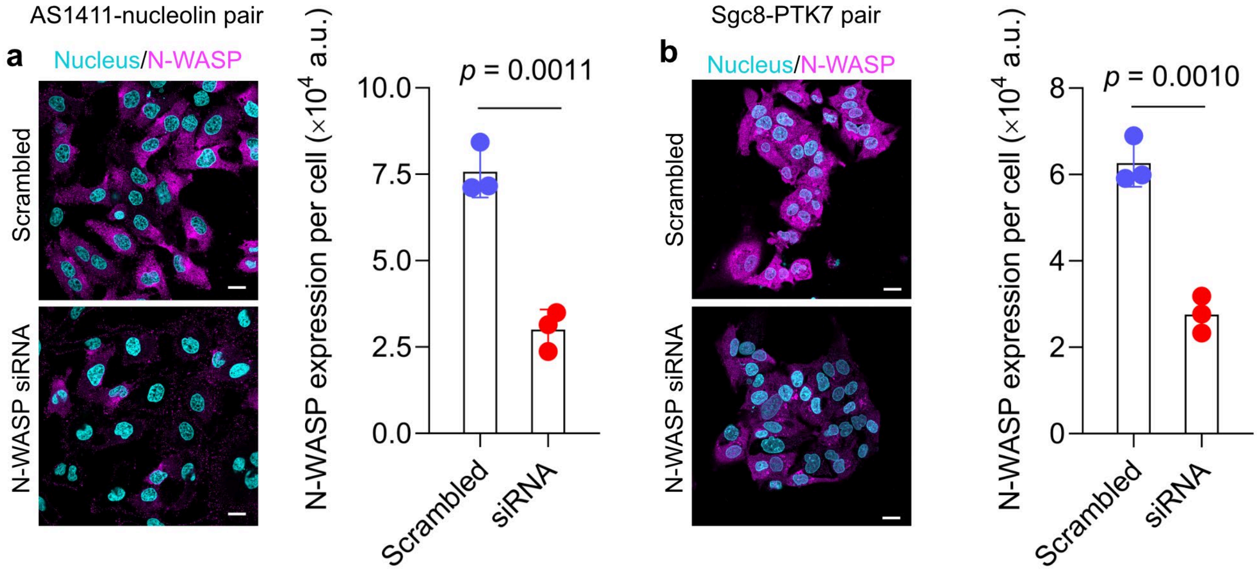

Supplementary Figure 10. **a** Representative immunostaining images and quantification of N-WASP expression level of HeLa cells after siRNA knockdown. HeLa cells transfected with either scrambled or N-WASP siRNA were stained with anti-N-WASP primary antibody followed by Alexa Fluor™ 647-conjugated secondary antibody. n = 1372 and 1304 cells from 3 replicates for scrambled and siRNA groups. The image is representative of 3 replicates. Statistics: unpaired two-tailed Student's *t*-test. **b** Representative immunostaining images and quantification of N-WASP expression level of HepG2 cells after siRNA knockdown. HepG2 cells transfected with either scrambled or N-WASP siRNA were stained with anti-N-WASP primary antibody followed by Alexa Fluor™ 647-conjugated secondary antibody. n = 1222 and 1198 cells from 3 replicates for scrambled and siRNA groups. The image is representative of 3 replicates. Statistics: unpaired two-tailed Student's *t*-test. Both graphs are presented as mean ± s.d. a.u., arbitrary units. Scale bars = 20 µm. Source data for this figure is available in the Source Data file.

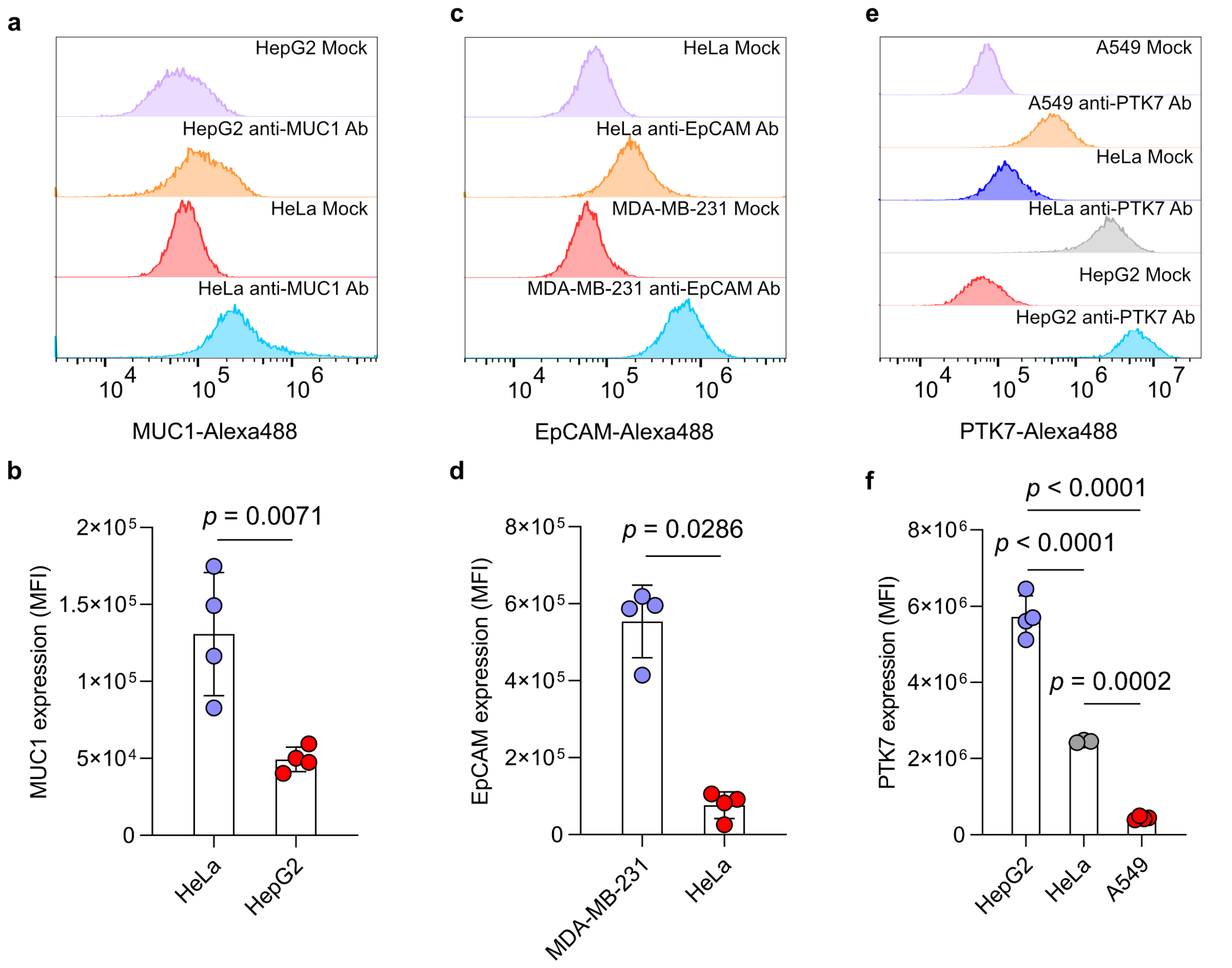

Supplementary Figure 11. a Representative flow cytometry histogram of HeLa and HepG2 cells stained with Alexa Fluor™ 488-conjugated anti-MUC1 primary. The image is representative of 4 replicates. **b** Quantification of mucin-1 expression levels on HeLa and HepG2 cells based on median fluorescence intensity (MFI). n = 4 replicates. Statistics: unpaired two-tailed Student's *t*-test. **c** Representative flow cytometry histogram of MDA-MB-231 and HeLa cells stained with Alexa Fluor™ 488-conjugated anti-EpCAM primary. The image is representative of 4 replicates. **d** Quantification of EpCAM expression levels on MDA-MB-231 and HeLa cells based on median fluorescence intensity. n = 4 replicates. Statistics: two-tailed, Mann-Whitney test. **e** Representative flow cytometry histogram of HepG2, HeLa, and A549 cells stained with anti-PTK7 primary antibody followed by Alexa Fluor™ 488-conjugated secondary antibody. Mock controls represent unstained cells, indicating cellular autofluorescence. The image is representative of 3 replicates. **f** Quantification of PTK7 expression levels on HepG2, HeLa, and A549 cells based on median fluorescence intensity. n = 4, 3, 4 replicates for HepG2, HeLa and A549, respectively. Statistics: One-way ANOVA with Bonferroni post-hoc tests. All graphs are presented as mean ± s.d. Source data for this figure is available in the Source Data file.

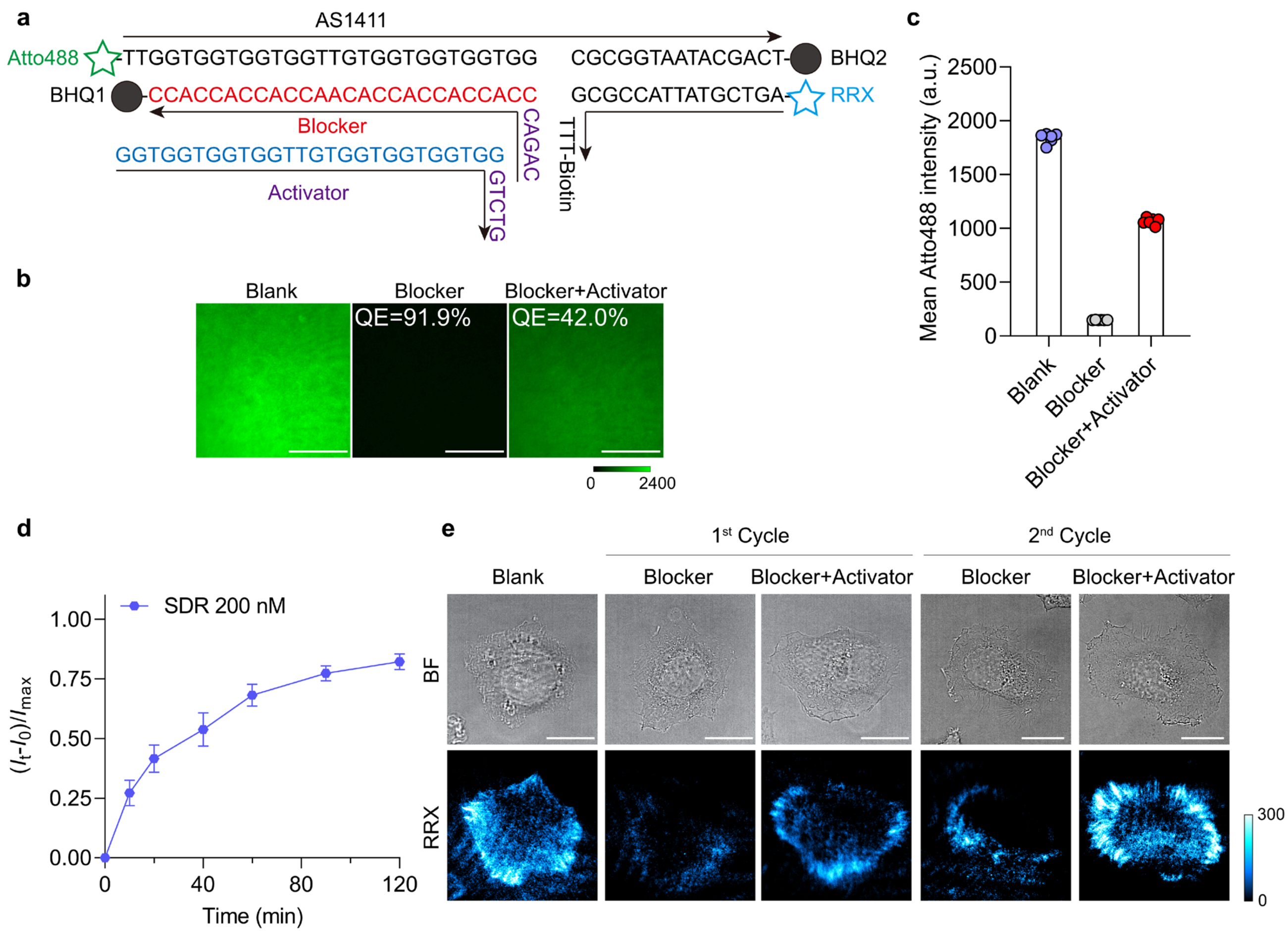

Supplementary Figure 12. a Detailed sequences for controlling AS1411 MPs by SDRs. **b-c** Fluorescence images (**b**) and corresponding quantification (**c**) showing blocking and reactivation of the surface-immobilized AS1411 MP. MPs are labeled with Atto488 at the 5' end. Incubation with 200 nM 3'-BHQ1–labeled blocker strands for 30 min results in a 91.9% decrease in Atto488 fluorescence. Subsequent medium exchange with the equimolar activator restores fluorescence by 49.9% within 30 min. Reaction medium: 1 % FBS, 1 % P/S, DMEM. n = 6 from 3 replicates. The image is representative of 3 replicates. **d** Plot of normalized surface Atto488 intensity versus time to compare the reconfiguration rate of AS1411 aptamer in SDR module. Surface-immobilized AS1411 MPs are initially blocked with DNA blocker and subsequently reconfigured by adding 200 nM DNA activator. At each reaction time point, the surface Atto488 intensity is normalized to that of unblocked AS1411 MPs labeled with Atto488, which serves as the positive control. Reaction medium: 1 % FBS, 1 % P/S, 100 µg/mL actin protein, DMEM. n = 15 from 3 replicates. **e** Representative brightfield and fluorescence images of HeLa cells on AS1411 MP surfaces after sequential blocker and activator treatment. The image is representative of 3 replicates. Both graphs are presented as mean ± s.d. a.u., arbitrary units. Scale bars = 20 µm. Source data for this figure is available in the Source Data file.

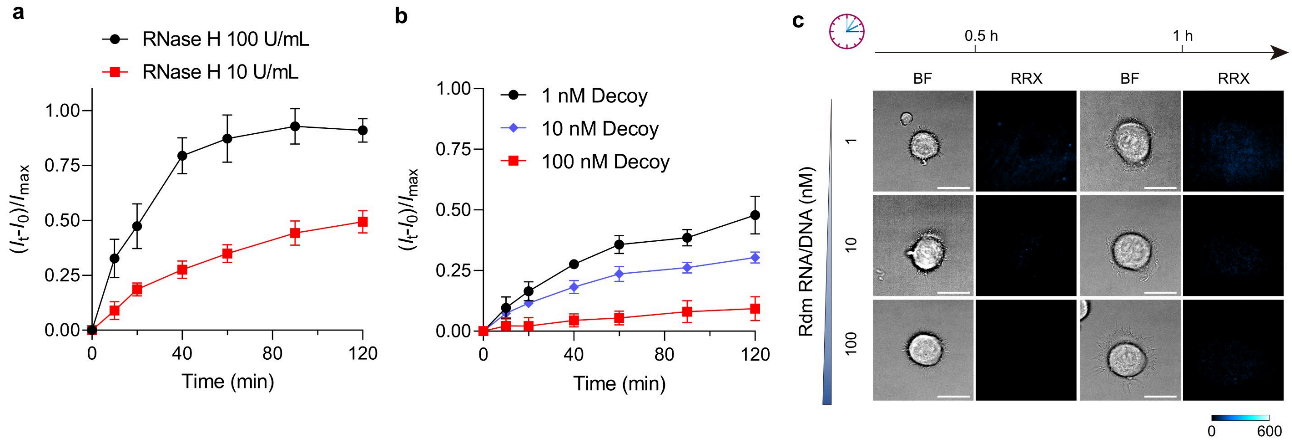

Supplementary Figure 13. a Plot of normalized surface Atto488 intensity versus time to compare the reconfiguration rate of AS1411 aptamer in RNA-RNase H module. Surface-immobilized AS1411 MPs are blocked with RNA blocker and degraded by RNase H at different concentrations (10 or 100 U/mL). At each reaction time point, the surface Atto488 intensity is normalized to that of unblocked AS1411 MPs labeled with atto488, which serves as the positive control. Reaction medium: 1 % FBS, 1 % P/S, 100 µg/mL actin protein, DMEM. n = 15 from 3 replicates. **b** Plot of normalized surface Atto488 intensity versus time to compare the reconfiguration rate of AS1411 aptamer in RNA-RNase H module in the presence of decoy strands. Surface-immobilized AS1411 MPs are blocked with RNA blocker and degraded by RNase H at 10 or 100 U/mL and decoy strands at 1-100 nM. Reaction medium: 1 % FBS, 1 % P/S, 100 µg/mL actin protein, DMEM. n = 15 from 3 replicates. At each reaction time point, the surface Atto488 intensity is normalized to that of unblocked AS1411 MPs labeled with atto488, which serves as the positive control. **c** Representative brightfield and fluorescence images of HeLa cells on RNA-blocked AS1411 MP surfaces after adding 10 U/mL RNase H and varying RNA/DNA duplex concentrations. The image is representative of 3 replicates. Both graphs are presented as mean ± s.d. Scale bars = 20 µm. Source data for this figure is available in the Source Data file.

Supplementary References

1. Sethi, S. *et al.* Nuclease-Resistant L-DNA Tension Probes Enable Long-Term Force Mapping of Single Cells and Cell Consortia. *Angew. Chem. Int. Ed.* **63**, e202413983 (2024).
