# Supplementary material for: Synthetic aptamer mechanoreceptors enable cell-specific force sensing and temporal control via DNA circuits
Source: Nat Commun. 2026 Mar 15;17:2492. doi: 10.1038/s41467-026-70765-w (PMC12992700; doi:10.1038/s41467-026-70765-w)
Supplement: Supplementary file 3 — Reporting Summary [file 41467_2026_70765_MOESM3_ESM.pdf]

nature portfolio

Corresponding author(s): Andreas Walther

Last updated by author(s): Feb 20, 2026

Reporting Summary

Nature Portfolio wishes to improve the reproducibility of the work that we publish. This form provides structure for consistency and transparency in reporting. For further information on Nature Portfolio policies, see our Editorial Policies and the Editorial Policy Checklist.

Statistics

For all statistical analyses, confirm that the following items are present in the figure legend, table legend, main text, or Methods section.

n/a	Confirmed	
☐	☒	The exact sample size (*n*) for each experimental group/condition, given as a discrete number and unit of measurement
☐	☒	A statement on whether measurements were taken from distinct samples or whether the same sample was measured repeatedly
☐	☒	The statistical test(s) used AND whether they are one- or two-sided<br>*Only common tests should be described solely by name; describe more complex techniques in the Methods section.*
☒	☐	A description of all covariates tested
☐	☒	A description of any assumptions or corrections, such as tests of normality and adjustment for multiple comparisons
☐	☒	A full description of the statistical parameters including central tendency (e.g. means) or other basic estimates (e.g. regression coefficient) AND variation (e.g. standard deviation) or associated estimates of uncertainty (e.g. confidence intervals)
☐	☒	For null hypothesis testing, the test statistic (e.g. *F*, *t*, *r*) with confidence intervals, effect sizes, degrees of freedom and *P* value noted<br>*Give P values as exact values whenever suitable.*
☒	☐	For Bayesian analysis, information on the choice of priors and Markov chain Monte Carlo settings
☒	☐	For hierarchical and complex designs, identification of the appropriate level for tests and full reporting of outcomes
☒	☐	Estimates of effect sizes (e.g. Cohen's *d*, Pearson's *r*), indicating how they were calculated

Our web collection on statistics for biologists contains articles on many of the points above.

Software and code

Policy information about availability of computer code

Data collection	Zeiss Elyra 7 Imaging System was used for microscopy image acquisition. NovoExpress v1.6.0 was used for flow data collection. Leica Stellaris5 Microscope (LAS X, v4.3.0.24308) was used for CLSM imaging.
Data analysis	Fiji was used for image analysis. FlowJo v10.8.1. was used for flow data analysis. Statistical results were analyzed using GraphPad Prism 8.0. All numerical data was visualized with Origin 2018, b9.5.0.193, Academic (OriginLab) or GraphPad Prism 8.0.

For manuscripts utilizing custom algorithms or software that are central to the research but not yet described in published literature, software must be made available to editors and reviewers. We strongly encourage code deposition in a community repository (e.g. GitHub). See the Nature Portfolio guidelines for submitting code & software for further information.

Data

Policy information about availability of data

All manuscripts must include a data availability statement. This statement should provide the following information, where applicable:

- Accession codes, unique identifiers, or web links for publicly available datasets
- A description of any restrictions on data availability
- For clinical datasets or third party data, please ensure that the statement adheres to our policy

Source data are provided with this paper.

Research involving human participants, their data, or biological material

Policy information about studies with human participants or human data. See also policy information about sex, gender (identity/presentation), and sexual orientation and race, ethnicity and racism.

Reporting on sex and gender	N/A
Reporting on race, ethnicity, or other socially relevant groupings	N/A
Population characteristics	N/A
Recruitment	N/A
Ethics oversight	N/A

Note that full information on the approval of the study protocol must also be provided in the manuscript.

Field-specific reporting

Please select the one below that is the best fit for your research. If you are not sure, read the appropriate sections before making your selection.

☒ Life sciences ☐ Behavioural & social sciences ☐ Ecological, evolutionary & environmental sciences

For a reference copy of the document with all sections, see nature.com/documents/nr-reporting-summary-flat.pdf

Life sciences study design

All studies must disclose on these points even when the disclosure is negative.

Sample size	No sample size calculation was performed. Each experiment was performed at least three independent times. For cell imaging, 10-24 images were captured in each replicate. For flow cytometry experiments, about 10,000 cells were recorded before gating. These sample sizes are sufficient to represent the experimental results.
Data exclusions	No data was excluded from the analyses
Replication	All experiments involving cells and/or DNA functionalized surfaces were repeated at least 3 times with different batches of surfaces, cells at different passage numbers to ensure reproducibility.
Randomization	No randomization was used, since all the experiments were done independently with several replicates.
Blinding	No group allocation was performed hence no blinding was used in the study.

Reporting for specific materials, systems and methods

We require information from authors about some types of materials, experimental systems and methods used in many studies. Here, indicate whether each material, system or method listed is relevant to your study. If you are not sure if a list item applies to your research, read the appropriate section before selecting a response.

Materials & experimental systems

n/a	Involved in the study	
☐	☒	Antibodies
☐	☒	Eukaryotic cell lines
☒	☐	Palaeontology and archaeology
☒	☐	Animals and other organisms
☒	☐	Clinical data
☒	☐	Dual use research of concern
☒	☐	Plants

Methods

n/a	Involved in the study	
☒	☐	ChIP-seq
☐	☒	Flow cytometry
☒	☐	MRI-based neuroimaging

Antibodies

Antibodies used	Anti-PTK7 monoclonal antibody (Thermo Fisher, MA5-25774, OTI2E7), Alexa Fluor 488 anti-CD227 (Mucin-1) monoclonal antibody (Thermo Fisher, 53-9893-82, SM3), Alexa Fluor 488 anti-CD326 (EpCAM) monoclonal antibody (Thermo Fisher, 53-8326-42, MH99),

	and Alexa Fluor 488 goat anti-mouse IgG (H+L) cross-adsorbed secondary antibody (Thermo Fisher, A-11001). Alexa Fluor™ 647 goat anti-mouse IgG (H+L) highly cross-adsorbed secondary antibody (Thermo Fisher, A-21236). N-WASP antibody (Santa Cruz, C-1, sc-271484)
Validation	Validation of anti-PTK7 monoclonal antibody (Thermo Fisher, MA5-25774, OTI2E7) can be found in: https://www.thermofisher.com/antibody/product/PTK7-Antibody-clone-OTI2E7-Monoclonal/MA5-25774.<br>Validation of Alexa Fluor 488 anti-CD227 (Mucin-1) monoclonal antibody (Thermo Fisher, 53-9893-82, SM3) can be found in: https://www.thermofisher.com/antibody/product/CD227-Mucin-1-Antibody-clone-SM3-Monoclonal/53-9893-82.<br>Validation of Alexa Fluor 488 anti-CD326 (EpCAM) monoclonal antibody (Thermo Fisher, 53-8326-42, MH99) can be found in: https://www.thermofisher.com/antibody/product/CD326-EpCAM-Antibody-clone-MH99-Monoclonal/53-8326-42.<br>Validation of Alexa Fluor 488 goat anti-mouse IgG (H+L) cross-adsorbed secondary antibody (Thermo Fisher, A-11001) can be found in: https://www.thermofisher.com/antibody/product/Goat-anti-Mouse-IgG-H-L-Cross-Adsorbed-Secondary-Antibody-Polyclonal/A-11001.<br>Validation of Alexa Fluor™ 647 goat anti-mouse IgG (H+L) highly cross-adsorbed secondary antibody can be found in: https://www.thermofisher.com/antibody/product/Goat-anti-Mouse-IgG-H-L-Highly-Cross-Adsorbed-Secondary-Antibody-Polyclonal/A-21236.<br>Validation of N-WASP antibody can be found in: https://www.scbt.com/p/n-wasp-antibody-c-1.

Eukaryotic cell lines

Policy information about cell lines and Sex and Gender in Research

Cell line source(s)	HeLa (ACC 57) and HepG2 (ACC 180) were purchased from DSMZ. A549 (DSMZ, ACC 107) and MDA-MB-231 (DSMZ, ACC 732) were gifts from Tanja Weil laboratory at Max Planck Institute for Polymer Research.
Authentication	Cell lines have not been authenticated.
Mycoplasma contamination	All cell lines are tested mycoplasma negative.
Commonly misidentified lines (See ICLAC register)	No misidentified cell lines were used

Plants

Seed stocks	N/A
Novel plant genotypes	N/A
Authentication	N/A

Flow Cytometry

Plots

Confirm that:

☒ The axis labels state the marker and fluorochrome used (e.g. CD4-FITC).

☒ The axis scales are clearly visible. Include numbers along axes only for bottom left plot of group (a 'group' is an analysis of identical markers).

☒ All plots are contour plots with outliers or pseudocolor plots.

☒ A numerical value for number of cells or percentage (with statistics) is provided.

Methodology

Sample preparation	Cells were detached using trypsin and was washed and injected into FlowCytometer. See Methods section for further details.
Instrument	Novocyte Quanteon (Agilent) with 4 excitation lasers (violet 405 nm, blue 488 nm, yellow-green 561 nm and red 640 nm) and 16 fluorescence detectors.
Software	NovoExpress v1.6.0, FlowJo v10.8.1.
Cell population abundance	At least 10,000 events were collected for each samples.

Gating strategy	Shown in Supplementary figure 3a.

☒ Tick this box to confirm that a figure exemplifying the gating strategy is provided in the Supplementary Information.
